# Accuracy of Ex-vivo Fluorescence Confocal Microscopy in Margin Assessment of Solid Tumors: A Systematic Review

**DOI:** 10.1369/00221554231212948

**Published:** 2023-11-15

**Authors:** Matthew Au, Ricardo Almeida-Magana, Tarek Al-Hammouri, Aiman Haider, Greg Shaw

**Affiliations:** Department of Targeted Intervention, University College London, London, United Kingdom, University College London Hospitals, London, United Kingdom; Department of Targeted Intervention, University College London, London, United Kingdom, University College London Hospitals, London, United Kingdom; Department of Urology, University College London Hospitals, London, United Kingdom; Department of Pathology, University College London Hospitals, London, United Kingdom; Department of Targeted Intervention, University College London, London, United Kingdom, University College London Hospitals, London, United Kingdom; Department of Urology, University College London Hospitals, London, United Kingdom

**Keywords:** brain neoplasms, breast neoplasms, confocal microscopy, margins of excision, prostatic neoplasms

## Abstract

Fluorescence confocal microscopy (FCM) is a novel technology that enables rapid high-resolution digital imaging of non-formalin-fixed tissue specimens and offers real-time positive surgical margin identification. In this systematic review, we evaluated the accuracy metrics of ex vivo FCM for intraoperative margin assessment of different tumor types. A systematic search of MEDLINE via PubMed, Embase, Cochrane Central Register of Controlled Trials, Web of Science, and Scopus was performed for relevant papers (PROSPERO ID: CRD42022372558). We included 14 studies evaluating four types of microscopes in six different tumor types, including breast, prostate, central nervous system, kidney, bladder, and conjunctival tumors. Using the Quality Assessment of Diagnostic Accuracy Studies tool, we identified a high risk of bias in patient selection (21%) and index test (36%) of the included studies. Overall, we found that FCM has good accuracy metrics in all tumor types, with high sensitivity and specificity (>80%) and almost perfect concordance (>90%) against final pathology results. Despite these promising findings, the quality of the available evidence and bias concerns highlight the need for adequately designed studies to further define the role of ex vivo FCM in replacing the frozen section as the tool of choice for intraoperative margin assessment:

## Introduction

A positive surgical margin (PSM) denotes the presence of residual cancer cells at the margin of resected specimens, and has been shown to increase the risk of local recurrence and decrease overall survival in several cancer types.^1–4^ Numerous studies have further stratified recurrence risk levels based on margin status, with the recommendation of additional therapies if a PSM is present.^5–8^ Accurate identification of PSMs during surgery is, therefore, crucial to guide secondary resection and avoid the need for reoperations or adjuvant treatment.

Frozen section (FS) is currently the standard method for intraoperative diagnosis and margin assessment in surgical oncology. However, it has limited sensitivity because only sections that are most likely to be representative are analyzed.^9^ If the entire surface of the specimen was analyzed using FS, the procedure would be too labor-, staff-, and time-intensive. In addition, incomplete cutting, tissue folding, distorted architecture, and freezing artifacts may occur.^10^ If the slides are not interpretable, resampling may be requested, further delaying the results. These drawbacks have restricted the adoption of routine FS in many centers. Moreover, analyzing small samples may result in tissue loss, compromising the accuracy of the formalin-fixed paraffin-embedded analysis (FFPE).

Fluorescence confocal microscopy (FCM), also known as confocal laser microscopy, is a novel imaging technique that produces high-resolution images of fresh specimens with cellular-level detail using photoreactive dyes. Compared with FS, FCM has the advantage of generating digital scans in minutes, requiring fewer consumables and minimal tissue processing. In addition, FCM can be placed within the operating room, avoiding the need to transport samples across sites. Although FCM is now routinely used in dermatology,^11^ interest in using FCM to guide surgical resection has increased in recent years. However, we realized a systematic review on this topic is lacking. The primary objective of this article is to summarize the current knowledge on ex-vivo FCM accuracy metrics in assessing the margin status of surgical specimens of non-skin solid tumors.

## Methods

This systematic review was conducted in accordance with the Preferred Reporting Items for Systematic Reviews and Meta-analyses Protocol (PRISMA-P) and the Cochrane Handbook for Systematic Reviews on Diagnostic Test Accuracy.^12,13^ Before conducting the review, a study protocol was developed and registered on the PROSPERO database (ID: CRD42022372558), outlining the search strategy, inclusion criteria, and risk of bias measures.

### Search Strategy

An electronic search was performed on MEDLINE via PubMed, Embase, Cochrane Central Register of Controlled Trials (CENTRAL), Web of Science, and Scopus. The search strategy is available in the Appendix.

### Eligibility

Study screening, data extraction, and risk of bias assessment were performed using the automated screening Covidence^14^ developed by the Cochrane Collaboration. Two independent reviewers (M.A. and R.A.-M.) screened the titles and abstracts, as well as the full texts of potentially eligible studies. Both randomized and non-randomized studies were included. Abstracts presented at conferences were included, whereas case reports (with two or fewer patients) and non-English papers were excluded. Non-peer-reviewed articles, editorial features, and news stories were also excluded. No time restrictions on the date of publication were used, and ongoing and unpublished studies were not included.

### Study Population and Target Condition

Studies including all types of cancer, except skin cancer, were eligible. Studies performed in animal models were excluded.

### Intervention

Patients who underwent surgical resection were included. Only analysis of the resected specimen was included, whereas evaluation of biopsies was excluded.

### Index Test

All types of ex vivo FCMs were included in this study, whereas studies evaluating in vivo handheld probe-based devices designed for endomicroscopic use were excluded unless the tissue was evaluated in an ex vivo setting. Other optical laser microscopies such as optical coherence tomography (OCT), Raman spectroscopy, near-infrared spectroscopy, photoacoustic microscopy, and narrow band imaging were all excluded.

### Reference Standard

Definitive histopathology report of the tumor status or the clinical routine (FFPE and hematoxylin and eosin-stained permanent section).

### Outcomes

Studies reporting the accuracy of ex-vivo FCM were included. Only studies reporting a binary outcome were considered, detecting the presence of PSMs or the presence of a tumor detected by the evaluator. Studies with only diagnostic or qualitative outcomes were excluded.

### Data Extraction

Data extraction was performed by four reviewers (M.A., R.A.-M., T.A.-H., and G.S.), who extracted the following information: study title, author, year of publication, country, full-text availability, study design, type of confocal microscope evaluated, type of control, blinding, profession of evaluator(s), number of evaluator(s), type of tumor, number of patients, number of images, number of PSMs identified by FCM and histopathology, sensitivity, specificity, and the rate of concordance between the index test and the reference standard (Cohen’s kappa [κ]-agreement).

### Quality Assessment of Included Studies

As recommended by the Cochrane Handbook for Systematic Reviews of Diagnostic Test Accuracy,^15^ we used the Quality Assessment of Diagnostic Accuracy Studies (QUADAS-2) tool^16^ to assess the risk of bias of included studies. Items were individually tailored for use in this review. The quality assessment involved four domains: patient selection, index test, reference standard, and flow and timing. Each domain included three questions, with two signaling questions and one risk of bias assessment. Signaling questions could be answered as “Yes,” “No,” or “Unclear,” whereas the risk of bias and concerns for applicability could be rated as “High,” “Low,” or “Unclear.” The results of the risk of bias assessment and applicability concerns were used to inform the strength of the evidence. The template for the risk of bias assessment is available in the Appendix.

### Statistical Analysis

As per the protocol, we planned a meta-analysis of accuracy results to be performed if more than three eligible studies in a particular tumor type were retrieved and the same outcome measures were reported. Forest plots of sensitivity and specificity, pooled sensitivity and specificity, and inconsistency would be calculated from the data retrieved, using a binary covariate model. In addition, a funnel plot would be constructed to test for publication bias.

However, due to the limited number of valid and applicable studies retrieved, the authors reached a consensus not to perform a meta-analysis. Only two studies on breast and two on prostate had low applicability concerns and risk of bias, so summary estimates were not presented, in accordance with the recommendations from the Cochrane Handbook for Systematic Reviews of Diagnostic Test Accuracy.^17^

## Results

### Study Selection

**Figure 1.**
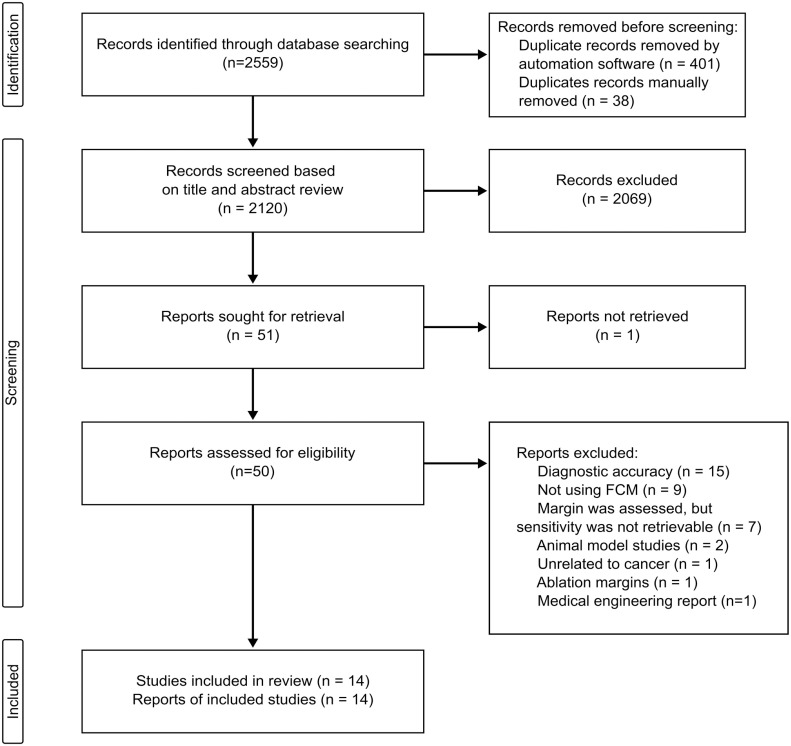
Preferred Reporting Items for Systematic Reviews and Meta-Analyses 2020 flow diagram. Abbreviation: FCM, fluorescence confocal microscopy.

We conducted a comprehensive database search on December 23, 2022. Figure 1 shows the PRISMA flow diagram. Initially, 2559 studies were identified, and after removing duplicates, 2120 titles were retained for screening. Of these, 50 full-text studies were assessed for eligibility, with 14 eventually meeting our inclusion criteria for data extraction. We were unable to retrieve the full text for one study.

### Excluded Studies

Of the 36 studies that underwent full-text assessment, we excluded 15 that focused on diagnostic accuracy for cancer subtypes rather than margin assessment. Nine studies did not meet our inclusion criteria for the index test, and seven evaluated margin status but lacked retrievable sensitivity and specificity data. Of these, four were abstracts from medical conferences. We also excluded two studies that used animal models, and three others that were unrelated to cancer or focused on ablation margins or medical device engineering.

### Quality of the Studies

**Figure 2.**
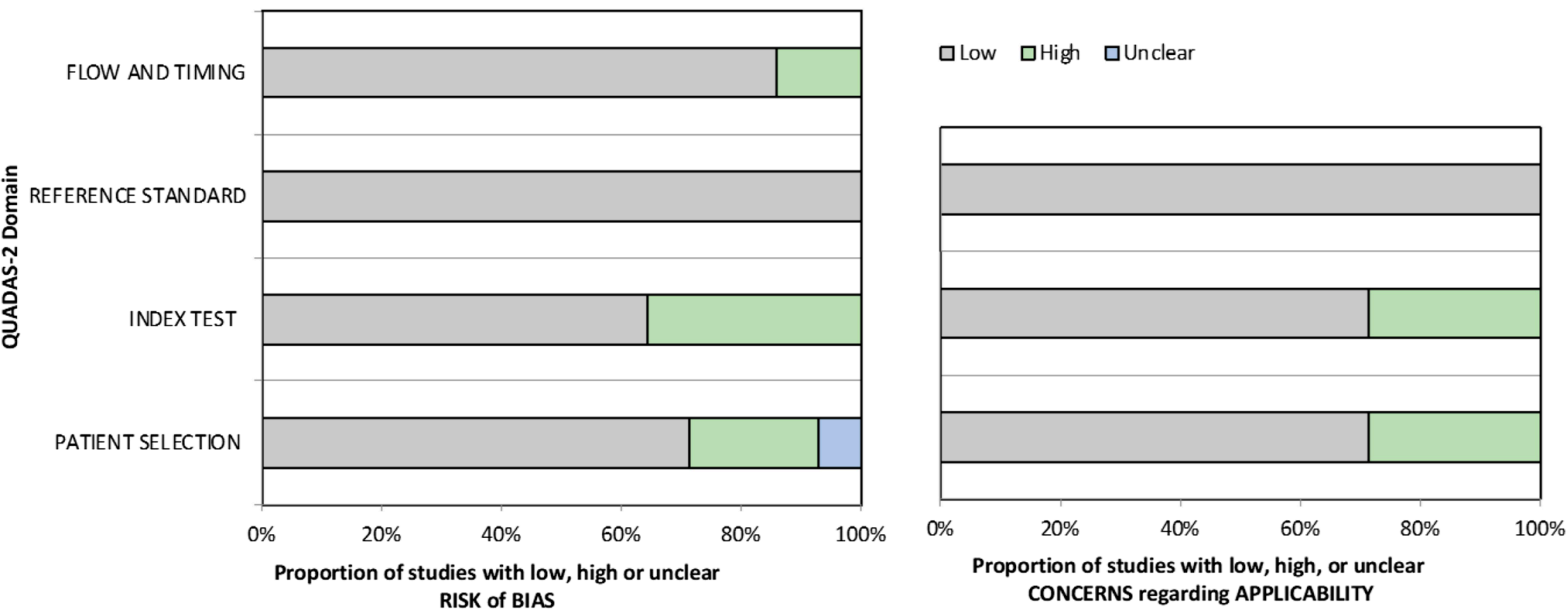
Risk of bias and applicability concerns graph. Abbreviation: QUADAS-2, Quality Assessment of Diagnostic Accuracy Studies.

Our risk of bias and applicability assessment is presented in Tables 1 and 2 and Fig. 2. We found a high risk of bias in patient selection (21%), index test assessment (36%), and flow and timing (14%). We also identified applicability concerns in patient selection (29%) and index test (29%) because some studies focused on diagnostic accuracy rather than margin evaluation, despite including evaluations of tumor present at the margin. Although we included these studies in our analysis for data completeness, we acknowledge that the lack of healthy tissue as a comparator in some studies remains a concern. In addition, some of the studies included in our analysis were retrospective case series and feasibility studies, which raises concerns about blinding strategies and patient selection criteria.

**Table 1.** Results of QUADAS-2 Risk of Bias Assessment of the Included Studies.

Reference	Tumor Type	Patient Selection	Index Test	Reference Standard	Flow and Timing
Golatta et al.^18^	Breast	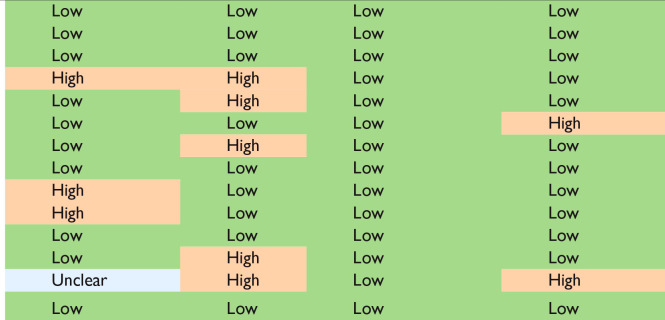			
Nackenhorst et al.^19^	Breast				
Sandor et al.^20^	Breast				
Scimone et al.^21^	Breast				
Baas et al.^22^	Prostate				
Rocco et al.^23^	Prostate				
Rocco et al.^24^	Prostate				
Rocco et al.^25^	Prostate				
Acerbi et al.^26^	Brain				
Belykh et al.^27^	Brain				
Martirosyan et al.^28^	Brain				
Esperto et al.^29^	Bladder				
Vreuls et al.^30^	Kidney				
Iovieno et al.^31^	Eye				
Count		Patient Selection	Index Test	Reference Standard	Flow and Timing
Low		10	9	14	12
High		3	5	0	2
Unclear		1	0	0	0

Abbreviation: QUADAS-2, Quality Assessment of Diagnostic Accuracy Studies. Green: Low risk, Red: High risk, Blue: Unclear.

**Table 2.** Results of Applicability Concern Assessment of the Included Studies.

Reference	Tumor Type	Patient Selection	Index Test	Reference Standard
Golatta et al.^18^	Breast	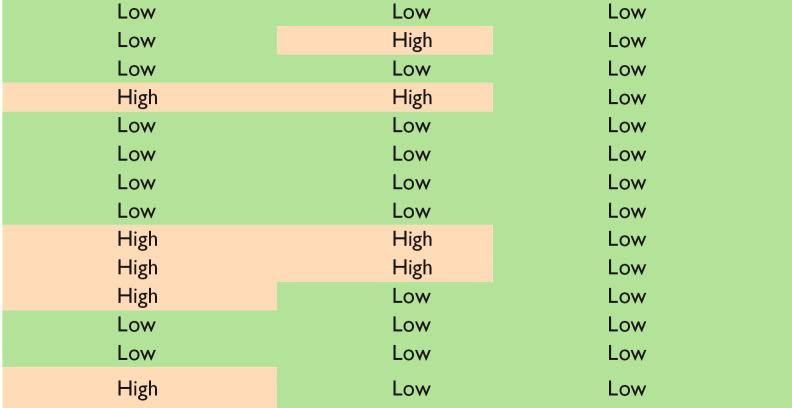		
Nackenhorst et al.^19^	Breast			
Sandor et al.^20^	Breast			
Scimone et al.^21^	Breast			
Baas et al.^22^	Prostate			
Rocco et al.^23^	Prostate			
Rocco et al.^24^	Prostate			
Rocco et al.^25^	Prostate			
Acerbi et al.^26^	Brain			
Belykh et al.^27^	Brain			
Martirosyan et al.^28^	Brain			
Esperto et al.^29^	Bladder			
Vreuls et al.^30^	Kidney			
Iovieno et al.^31^	Eye			
Count		Patient Selection	Index Test	Reference Standard
Low		10	10	14
High		4	4	0
Unclear		0	0	0

Legend: Green: Low risk, Red: High risk.

### Results of the Included Studies

Fourteen studies were included in this systematic review, evaluating breast cancer,^18–31^ prostate cancer,^20,27^ brain tumors,^26–28^ kidney,^29^ bladder,^30^ and conjunctival tumors.^31^ Of these, six studies used the VivaScope FCM (München, Germany), four used the Histolog FCM (Lausanne, Switzerland), three used the Carl Zeiss FCM (Jena, Germany), and one used a multimodal microscope that combined fluorescence microscopy, OCT, and FCM. Tables 3 and 4 summarizes the accuracy measurements of the included studies. However, 95% confidence intervals are not presented, as they were not reported in all but two studies.^20,27^

**Table 3.** Characteristics of the Included Studies.

Reference	Country	Tumor Type	Microscope Used	No. of Patients	No. of Specimens	No. of Images Generated	No. of PSMs Detected by FCM (and Reported by Histopathology Report)
Golatta et al.^18,d^	Germany	Breast	Histolog	50	50	300	48 of 300 images (48 of 300)
Nackenhorst et al.^19^	Austria	Breast	VivaScope	13	13	NR	1 of 13 specimens (2 of 13)
Sandor et al.^20^	Germany	Breast	Histolog	40	40	240	Not reported (13 of 40)
Scimone et al.^21^	USA	Breast	Multimodal^a^	NR	20	NR	10 of 20 specimens (8 of 20)
Baas et al.^22^	Netherlands	Prostate	Histolog	50	50	96	15 of 96 images (14 of 96)
Rocco et al.^23,d^	Italy	Prostate	VivaScope	11	16	16	0 of 11 specimens (0 of 11)
Rocco et al.^24^	Italy	Prostate	VivaScope	8	8	36	1 of 8 specimens (1 of 8)
Rocco et al.^25^	Italy	Prostate	VivaScope	24	72	72	4 of 24 specimens (4 of 24)
Acerbi et al.^26^	Italy	Brain	CONVIVO	15	30	30^c^	5 of 15 specimens (14 of 15)
Belykh et al.^27^	USA	Brain	CONVIVO	47	122	122	Not reported (122 of 122)
Martirosyan et al.^28^	USA	Brain	CONVIVO^b^	106	106	1960	94 of 106 specimens (99 of 106)
Esperto et al.^29,d^	Italy	Bladder	VivaScope	15	44	44	Not reported
Vreuls et al.^30,d^	Netherlands	Kidney	Histolog	6	6	6	2 of 6 specimens (2 of 6)
Iovieno et al.^31^	Italy	Eye	VivaScope	16	16	32	3 of 16 specimens (3 of 16)

Abbreviations: PSM, positive surgical margin; FCM, fluorescence confocal microscopy; NR, not retrievable.

a Multimodal: Combined optical coherence tomography, fluorescence microscopy, and reflectance confocal microscopy.

b Zeiss LSM CONVIVO.

c Thirty biopsies were collected in total, 15 were analyzed by frozen section and 15 were by permanent section.

d Only abstract available.

**Table 4.** Diagnostic Performance of FCM in Margin Assessment Across Included Studies.

Reference	TP	TN	FP	FN	Sensitivity (%)	Specificity (%)	PPV	NPV	κ-Agreement (%)
Breast									
Golatta et al.^18^	48	252	0	0	100^a^	100^a^	100^a^	100^a^	100.0^a^
Nackenhorst et al.^19^	0	10	1	2	0^a^	91^a^	0^a^	83^a^	87.9^a^
Sandor et al.^20^	NR	NR	NR	NR	54	85	NR	NR	NR
Scimone et al.^21^	8	10	2	0	100^a^	83^a^	80^a^	100^a^	90.0^a^
Prostate									
Baas et al.^22^	12	79	3	2	86	96	80	98	79.7
Rocco et al.^23^	0	11	0	0	NA	100^a^	NA	100^a^	100.0^a^
Rocco et al.^24^	1	7	0	0	100^a^	100^a^	100^a^	100^a^	100.0^a^
Rocco et al.^25^	4	20	0	0	100^a^	100^a^	100^a^	100^a^	100.0^a^
Brain									
Acerbi et al.^26^	5	1	0	9	36^a^	100^a^	100^a^	10^a^	NR
Belykh et al.^27^	73	18	2	30	72	90	97	38	77.8^a^
Martirosyan et al.^28^	99	7	0	5	95^a^	100^a^	100^a^	58^a^	90.6^b^
Bladder									
Esperto et al.^29^	NR	NR	NR	NR	0.88	0.81	NR	NR	NR
Kidney									
Vreuls et al.^30^	2	4	0	0	100^a^	100^a^	100^a^	100^a^	100.0^a^
Eye									
Iovieno et al.^31^	3	13	0	0	100^a^	100^a^	100^a^	100^a^	100.0^a^

Abbreviations: TP, true positive; TN, true negative; FP, false positive; FN, false negative; PPV, positive predictive value; NPV, negative predictive value; κ, Cohen’s kappa coefficient; NR, not retrievable; NA, not applicable; FCM, fluorescence confocal microscopy.

a Extrapolated from the raw data provided from the article.

b Although concordance between tumor versus non-tumor is 100%, the value presented is the concordance between if margin is defined as tumor, infiltrative, or healthy tissues.

### Breast Cancer

The four included studies evaluated a total of 123 breast specimens.^18–21^ Two studies reported high accuracy levels (>90%) for margin assessment.^18,21^ Golatta et al.^18^ published an abstract on 50 patients, reporting a sensitivity of 45.8% for the Histolog scanner compared with 39.6% with routine clinical practice involving ultrasound (US), with more accurate information anticipated when the full paper is available. Scimone et al.^21^ studied a multimodal FCM in 20 specimens, reporting no false negatives and two false positives.

Sandor et al.^20^ reported a sensitivity of 54% and specificity of 85% for the detection of PSM using the Histolog scanner with an en-face technique. Seven PSMs were identified, suggesting a potential reduction in re-excision rates of 58.3% if surgery was guided using this methodology (15). In contrast, Nackenhorst et al.^19^ were unable to identify any true PSM due to the absence of ink on cross-sectional images. However, they report that adipose tissue architecture in FCM images was comparable to FFPE sections and superior to FS, making it an attractive alternative to process tissue rich in adipose cells.

### Prostate Cancer

A total of 146 prostate specimens were evaluated across four studies.^22–25^ The majority of studies demonstrated an almost perfect κ-agreement (>80%) between FCM and FFPE for assessing tumor margins, and no PSMs detected in one study.^23^

The group led by Rocco et al. contributed three of the included studies. In their initial study, they utilized the VivaScope microscope to analyze 11 specimens, all of which had negative margins and were concordant with FFPE.^23^ In their second study, they described a Mohs-like shaving technique and identified a PSM in one out of eight patients.^24^ In the last study, they analyzed 24 specimens, detecting four PSMs intraoperatively. After secondary resection, all 24 patients had negative final surgical margin status at the site adjacent to the neurovascular bundles.^24^ However, one patient was found to have a PSM outside the Mohs section imaged by FCM. Baas et al.^22^ evaluated the performance of the Histolog scanner using an en-face technique, and found a high sensitivity (86%) and specificity (96%) against FFPE, with a near perfect κ-agreement (79.7%). They also compared the technique against FS analysis (NeuroSAFE) and reported a κ-agreement of 80%.

### Central Nervous System Neoplasms

A total of 243 specimens were evaluated across three studies for brain tumors. One study used the desktop-based Zeiss LSM 710/5Live DUO system (Carl Zeiss Meditec, Oberkochen Germany), and two studies used the CONVIVO confocal laser endomicroscopy (CLE) system (Carl Zeiss Meditec, Oberkochen Germany). FCM exhibited a high specificity (ranging from 90% to 100%) for diagnosing neurological tumors, whereas sensitivity varied from 36% to 95%.^26–28^ Cohen’s κ-agreement on the presence of tumor was 77.8%-100%^27,28^ and not reported nor retrievable in one study.^26^

Martirosyan et al.^28^ classified any images showing any distinguishable tumor irrespective of subtyping as positive, whereas non-diagnostic images were classified as negative, resulting in a 94.9% sensitivity and 100.0% specificity. Acerbi et al.^26^ evaluated only glioblastoma specimens and because sensitivity and specificity were not reported, 66.7% of the biopsies taken at the margin were concordant with the FFPE. Belykh et al.^27^ took a different approach, and interpreted CLE images as lesional and non-lesional, with non-diagnostic images included in non-lesional. The prevalence of positive tumor diagnosed by FFPE was 84%, and the sensitivity and specificity achieved by a trained neuropathologist were 72% and 90%, respectively. Interestingly, the accuracy of surgeons to interpret CLE images was also assessed, and the experienced neurosurgeon performed slightly better with a sensitivity and specificity of 74% and 92%.

### Other Cancers

The studies included in our review evaluated surgical margins of bladder, kidney, and conjunctival tumors.^29–31^ Esperto et al.^29^ reported perfect concordance between FCM and FS, with high sensitivity (88%) and specificity (81%) for urethral margins when compared with the FFPE. Vreuls et al.^30^ investigated the use of FCM in partial nephrectomies and demonstrated perfect concordance (100%) on the presence of tumor, with both FCM and FFPE identifying two PSMs in six tumor specimens with sensitivity and specificity of 100%. Iovieno et al.^31^ evaluated 16 consecutive patients with conjunctival tumors correctly identifying three PSMs using FCM.

## Discussion

To our knowledge, this is the first systematic review to aggregate knowledge on the accuracy of ex-vivo FCM for assessing surgical margins in non-cutaneous solid tumors. Because our findings suggest that FCM is a promising tool with a high sensitivity, specificity, and concordance across most cancer types, it should be noted that most studies have small sample sizes, and caution is warranted due to the high risk of bias and absence of a priori power calculation. Moreover, given the diverse morphological features among tumor subtypes and differences in margin assessment across surgical specialties, we will discuss our findings separately on each tumor type.

### Breast Cancer

Clinical guidelines recommend secondary resection of the tumor bed when a PSM is found during breast conserving.^32^ A meta-analysis reported that cytology had the highest pooled sensitivity (91%) and specificity (95%), followed by FS (86% sensitivity, 96% specificity) and optical imaging (85% sensitivity, 87% specificity). Intraoperative US and radiography demonstrated low sensitivity (59% and 53%, respectively) and specificity (81% and 84%, respectively).^33^ The poor uptake of FS and cytology in routine practice is limited by slow turnaround time and logistical constraints.^33^ FCM has theoretical advantages that may overcome these limitations. Three of the included studies showed improved accuracy for diagnosing the presence of a PSM compared with FFPE.^18,19^ The multimodal bespoke microscope used in the study by Scimone et al.^21^ had the highest reported accuracy, although the interpretation of the results should be taken with care owing to the study being sponsored by the industry and high risk of bias in patient selection. Unfortunately, no other studies evaluating this platform were found.

Nevertheless, the use of FCM in evaluating margins is more advanced in breast cancer than in other tumor types, and several studies have reported that FCM can distinguish clear morphological features and potentially reduce re-operation rates. Future research should evaluate the impact of FCM on oncological outcomes and assess whether it can be incorporated as a new standard of care.

### Prostate Cancer

In radical prostatectomy, achieving oncological control while minimizing the risk of post-surgical complications such as incontinence and erectile dysfunction remains a challenge for surgeons.^34^ A significant barrier to performing nerve-sparing surgical dissection near the prostate is the concern regarding the extent of cancer involvement in the adjacent nerve bundles. NeuroSAFE, a standardized intraoperative FS technique, can address this risk.^35,36^ However, like FS in other tumor types, limitations of NeuroSAFE could be addressed by FCM. Previous studies have proven that pathologists can use FCM to reliably identify prostate cancer in biopsy cores.^22^ Our review confirms that FCM also has high accuracy when applied to margin evaluation of the posterolateral margins of the prostate.

Interestingly, Baas et al.^22^ identified that in patients undergoing secondary resection due to a PSM identified on NeuroSAFE, all PSMs of ≤5 mm were negative on the secondary resected specimens. This provides further evidence to strengthen the concept that small (<3 mm) PSMs may be irrelevant and have the same risk of biochemical recurrence as those with negative margins.^37–39^

It is important to highlight that we found several potential bias concerns. Blinding was not performed in one study,^24^ and interpretation was performed by a single pathologist in all studies. Furthermore, most studies had a small sample size and low prevalence of PSMs. In addition, the majority of the available studies were conducted by the same research team, which may limit the generalizability of the results.

### Central Nervous System Neoplasms

Neurosurgical treatment for central nervous system (CNS) neoplasms aims to achieve complete resection while preserving functional safety. However, due to the infiltrative nature of histological subtypes such as gliomas and the high risk of damaging vital brain structures, intraoperative margin assessment in neurosurgery differs from other tumor types. First, due to the associated side effects of removing neural tissue, the detection of PSMs relied on biopsies instead of evaluating the margin of resected specimens. Second, two of the studies used a handheld probe device with a small field of view designed for in-vivo scanning.^26,27^ Third, a high proportion of tumor under FFPE was found in these studies, likely due to sampling at the tumor margin rather than the resection margin; therefore, reported sensitivity and specificity need to be interpreted with caution. Fourth, intraoperative assessment during brain surgery has two components, providing differential diagnosis and identifying tumor at the margin; the definition of concordance may vary in neurosurgery where margins are defined as presence of tumor, infiltrating tumor, or healthy tissue.

Surprisingly, in the study by Belykh et al.,^27^ an experienced neurosurgeon trained in FCM image interpretation was able to diagnose Gliomas with high accuracy compared to an inexperienced neurosurgeon supported by a non-specialist pathologist. This finding supports the idea that the interpretation of confocal images may have a shorter learning curve and could be a reliable tool if the evaluator is adequately trained. Overall, FCM accuracy for guiding resections of CNS tumors appears promising. However, the available evidence is limited by the quality of the studies.

### Other Cancers

Esperto et al.^29^ and Vreuls et al.^30^ both presented their findings in bladder and renal cancer, respectively, as abstracts. However, we anticipate that more accurate and comprehensive results will be published upon the release of their full reports. Of note, Vreuls et al.^30^ mentioned that FCM could diagnose angiomyolipoma, but differentiating between types of renal cell carcinoma was not feasible.

Iovieno et al.^31^ were the first to conduct a prospective trial to investigate margin assessment using FCM. The study provided additional details about FCM’s morphological characteristics in different tissue samples. Unfortunately, FCM analysis in this study was not blinded.

### Different Types of Confocal Microscopes

Most breast cancer articles employed Histolog FCM, whereas VivaScope FCM was predominantly used in urology studies. Both microscopes are desktop-based systems. The VivaScope system employs a dual laser system, with a reflectance mode at a wavelength of 785 nm and fluorescence at 488 nm, enabling dual contrast and high-resolution images in various depths. In contrast, the Histolog FCM excites tissue fluorescence with a laser at 488 nm, and fluorescence emission with a wavelength of >500 nm is collected, producing a toluidine-blue monostain-like image. The scanner tray of Histolog FCM allows a larger scan area (48 × 36 mm), whereas VivaScope FCM provides a maximum total scan area of 25 × 25 mm and a higher vertical resolution of at least 4 μM with a magnification of ×550. However, VivaScope FCM takes up to 4 or 5 min per sample, sacrificing contrast and depth capabilities compared with the Histolog FCM which requires approximately 50 sec per image.

The CONVIVO system operates as a miniaturized confocal microscope that operates using a 488-nm wavelength blue laser light and provides a real-time field of view of 475 × 267 μM. The desktop Carl Zeiss FCM is the previous version of the CONVIVO system. Both microscopes have the advantage of fast image acquisition that can be transformed into a video, allowing both ex-vivo analysis and in-vivo analysis when applied as a handheld probe. However, the field of view restricts its use in larger samples.

Scimone et al. (Scimone, Krishnamurthy, Maguluri, Preda, Park, Grimble, Song, Ban and Iftimia, 2021)^21^ used a multimodal microscope that combines fluorescence microscopy, FCM, and OCT. The images acquired were processed by a semi-automated algorithm generating a series of en-face and cross-sectional images. The proof-of-concept study showed excellent sensitivity (100%) and specificity (83.3%), but no other studies have confirmed these findings. Further evaluation directly comparing the performance of each FCM is needed to determine the preferred brand specific to the tissue type.

### Limitations

Several limitations of this review must be acknowledged. First, due to the novelty of FCM as a technique, the literature lacks clinical trials with adequate power, and most of the studies identified aimed to demonstrate proof of concept. As a result, despite being planned in the protocol, conducting a meta-analysis was not feasible. In addition, some studies focused primarily on diagnostic accuracy rather than margin assessment, but they were included as we were able to retrieve or extrapolate FCM’s sensitivity and specificity from available results. Last, cancer subtypes were not further classified in our review, and because there are morphological differences between histological subtypes, the accuracy of FCM may vary, and the ability to detect the presence of tumor may be affected.

### Future Perspective

As interest in this technology grows, subsequent studies should be planned with adequate power calculations to provide narrow confidence interval estimates on the accuracy of this technique. To reduce the risk of evaluator bias, blinded assessment should be incorporated. In addition, studies should aim to explore FCM potential application in more tumor types, including those that are difficult to evaluate using FS. Furthermore, studies should also focus on exploring the cost-effectiveness of FCM and its potential impact on patient outcomes. To ensure consistency and comparability across studies, we encourage authors to consider following the (Standard for Reporting of Diagnostic Accuracy Studies) (STARD) guidelines for diagnostic accuracy in their reports.^40^

In conclusion, currently available evidence shows FCM has potential to facilitate margin assessment of multiple tumor types, given its apparent high diagnostic accuracy. FCM may enable more centers to perform real-time evaluation of PSMs and transform the pathology workflow. However, the current limitations in the evidence emphasize the need for more rigorously designed studies to confirm FCM diagnostic accuracy before it is incorporated in clinical practice.

## Appendix

### Search Strategy

The electronic databases and search strategy that will be included are as follows:

- MEDLINE (via PubMed)
  - (“Microscopy, Confocal”[Mesh] OR Fluorescence OR Laser OR Histolog OR VivaScope) AND “Margins of Excision”[Mesh]

- Embase
  - ((Histolog or VivaScope or Confocal) and (Surgical margin or margin of excision or resection margin)).af.

- CENTRAL
  - (Microscopy, Confocal):ti,ab,kw OR (VivaScope):ti,ab,kw OR (Histolog):ti,ab,kw AND (Margins of Excision):ti,ab,kw

- Web of Science
  - ((((TS=(confocal) OR TS=(con-focal) OR TS=(laser) OR TS=(laser-scanning)) AND (TS=(microscopies) OR TS=(microscopy)) OR TS=(histolog) OR TS=(vivascope)) AND (TS=(excision) OR TS=(resection) OR TS=(surgical) OR TS=(positive) OR TS=(negative) OR TS=(tumour-free) OR TS=(tumor-free) OR TS=(tumour) OR TS=(tumor) OR TS=(free) OR TS=(assessment)) AND (TS=(margin) OR TS=(margins)))

- Scopus
  - TITLE-ABS-KEY ((((confocal OR con-focal OR laser OR laser-scanning) AND (microscopies OR microscopy)) OR (histolog OR vivascope)) AND ((excision OR resection OR surgical OR positive OR negative OR tumour-free OR tumor-free OR tumour OR tumor OR free OR assessment) AND (margin OR margins))) AND (LIMIT-TO (DOCTYPE, “ar”) OR LIMIT-TO (DOCTYPE, “cr”))

### Quality Assessment

#### QUADAS Template

1. Patient selection 1

Could the selection of patients have introduced bias?

- Yes
- No
- Unclear

(Extractors will also be able to add supporting text to justify their judgments.)

2. Patient selection 2

Did the study avoid inappropriate exclusion?

- Yes
- No
- Unclear

(Extractors will also be able to add supporting text to justify their judgments.)

3. Patient selection 3

Are there any concerns that the included patients do not match the review question? Could the selection of patients have introduced bias (high = high risk of bias; low = low risk of bias)?

- High
- Low
- Unclear

(Extractors will also be able to add supporting text to justify their judgments.)

4. Index test 1

Were the FCM results interpreted without the knowledge of the results of the control?

- Yes
- No
- Unclear

(Extractors will also be able to add supporting text to justify their judgments.)

5. Index test 2

Could the interpretation of the FCM results have introduced bias?

- Yes
- No
- Unclear

(Extractors will also be able to add supporting text to justify their judgments.)

6. Index test 3

Are there concerns that the index test or its interpretation differs from the review question? Could the conduct or interpretation of the FCM results have introduced bias (high = high risk of bias; low = low risk of bias)?

- High
- Low
- Unclear

(Extractors will also be able to add supporting text to justify their judgments.)

7. Reference standard 1

Is the reference standard likely to correctly classify the target condition? (Is this reference standard performed by an adequately trained pathologist?)

- Yes
- No
- Unclear

(Extractors will also be able to add supporting text to justify their judgments.)

8. Reference standard 2

Were the reference standard results interpreted without the knowledge of the results of the index test (FCM)?

- Yes
- No
- Unclear

(Extractors will also be able to add supporting text to justify their judgments.)

9. Reference standard 3

Could the reference standard, its conduct, or its interpretation have introduced bias (high = high risk of bias; low = low risk of bias)?

- High
- Low
- Unclear

(Extractors will also be able to add supporting text to justify their judgments.)

10. Flow and timing 1

Did all patients receive a reference standard?

Did all patients receive the same reference standard?

- Yes
- No
- Unclear

(Extractors will also be able to add supporting text to justify their judgments.)

11. Flow and timing 2

Were all patients included in the analysis?

Are the proportions and reasons for missing data similar across index tests?

- Yes
- No
- Unclear

(Extractors will also be able to add supporting text to justify their judgments.)

12. Flow and timing 3

Could the patient flow have introduced bias (high = high risk of bias; low = low risk of bias)?

- High
- Low
- Unclear

(Extractors will also be able to add supporting text to justify their judgments.)

13. Other sources of bias

State any important concerns about bias not addressed in the other domains in the tool. If particular questions/entries were pre-specified in the review’s protocol, responses should be provided for each question/entry.

- Yes
- No
- Unclear

(Extractors will also be able to add supporting text to justify their judgments.)

#### Applicability Concerns

14. Patient selection

Are there any concerns in regard to the applicability to our research question?

- Yes
- No
- Unclear

(Extractors will also be able to add supporting text to justify their judgments.)

15. Index test

Are there any concerns in regard to the applicability to our research question?

- Yes
- No
- Unclear

(Extractors will also be able to add supporting text to justify their judgments.)

16. Reference standard
Are there any concerns in regard to the applicability to our research question?

- Yes
- No
- Unclear

(Extractors will also be able to add supporting text to justify their judgments.)
